# Using healthcare systems data for outcomes in clinical trials: issues to consider at the design stage

**DOI:** 10.1186/s13063-024-07926-z

**Published:** 2024-01-29

**Authors:** Alice-Maria Toader, Marion K. Campbell, Jennifer K. Quint, Michael Robling, Matthew R Sydes, Joanna Thorn, Alexandra Wright-Hughes, Ly-Mee Yu, Tom. E. F. Abbott, Simon Bond, Fergus J. Caskey, Madeleine Clout, Michelle Collinson, Bethan Copsey, Gwyneth Davies, Timothy Driscoll, Carrol Gamble, Xavier L. Griffin, Thomas Hamborg, Jessica Harris, David A. Harrison, Deena Harji, Emily J. Henderson, Pip Logan, Sharon B. Love, Laura A. Magee, Alastair O’Brien, Maria Pufulete, Padmanabhan Ramnarayan, Athanasios Saratzis, Jo Smith, Ivonne Solis-Trapala, Clive Stubbs, Amanda Farrin, Paula Williamson

**Affiliations:** 1 MRC-NIHR Trials Methodology Research Partnership, Department of Health Data Science, University of Liverpool, Liverpool, UK; 2 Health Services Research Unit, University of Aberdeen, Aberdeen, AB25 2ZD UK; 3 School of Public Health &, National Heart and Lung Institute, Imperial College London, London, UK; 4 Centre for Trials Research, Cardiff University, Cardiff, CF14 4YS UK; 5 MRC Clinical Trials Unit at UCL, Institute of Clinical Trials and Methodology, UCL, London, UK; 6 BHF Data Science Centre, Health Data Research UK, London, UK; 7 Health Economics Bristol, Population Health Sciences, University of Bristol, Bristol, UK; 8 Clinical Trials Research Unit, Leeds Institute of Clinical Trials Research,, University of Leeds, Leeds, LS2 9JT UK; 9 Oxford Primary Care CTU, Nuffield Department of Primary Care Health Sciences, University of Oxford, Oxford, UK; 10 William Harvey Research Institute, Queen Mary University of London, London, EC1M 6BQ UK; 11 Cambridge Clinical Trials Unit, Cambridge, UK; 12 Bristol Trials Centre, Population Health Sciences, Bristol Medical School, University of Bristol, Bristol, BS8 2PS UK; 13 UCL Great Ormond Street Institute of Child Health, London, WC1N 1EH UK; 14 Swansea Trials Unit, Swansea University, Swansea, SA2 8PP UK; 15 Barts Bone and Joint Health, Blizard Institute, Barts and The London School of Medicine and Dentistry, Queen Mary University of London, London, UK; 16 Pragmatic Clinical Trials Unit, Wolfson Institute of Population Health, Queen Mary University of London, London, E1 2AB UK; 17 Intensive Care National Audit & Research Centre, London, UK; 18 Manchester University NHS Foundation Trust, Manchester, UK; 19 Ageing and Movement Research Group, Bristol Medical School, University of Bristol, Bristol, UK; 20 Older People’s Unit, Royal United Hospitals NHS Foundation Trust, Bath, UK; 21 School of Medicine, University of Nottingham and Nottingham City Care Partnership, Nottingham, UK; 22 Department of Women and Children’s Health, King’s College London, London, UK; 23 Division of Medicine, UCL Institute for Liver and Digestive Health, Royal Free Campus, Upper 3Rd FloorRowland Hill Street, London, NW3 2PF UK; 24 Department of Surgery and Cancer, Imperial College London, London, W21PB UK; 25 Keele Clinical Trials Unit, Faculty of Medicine and Health Sciences, Keele University, Staffordshire, UK; 26 Birmingham Clinical Trials Unit (BCTU), Institute of Applied Health Research College of Medical and Dental Sciences, The University of Birmingham, Edgbaston, Birmingham, B15 2TT UK; 27 Department of Cardiovascular Sciences, University of Leicester, Leicester, UK; 28 Bristol Medical School, University of Bristol, Bristol, BS8 2PS UK; 29 Liverpool Clinical Trials Centre, University of Liverpool, Liverpool, UK

**Keywords:** Healthcare systems data, Outcomes, Clinical trials, Routinely collected data, Data validity, Registries

## Abstract

**Background:**

Healthcare system data (HSD) are increasingly used in clinical trials, augmenting or replacing traditional methods of collecting outcome data. This study, PRIMORANT, set out to identify, in the UK context, issues to be considered before the decision to use HSD for outcome data in a clinical trial is finalised, a methodological question prioritised by the clinical trials community.

**Methods:**

The PRIMORANT study had three phases. First, an initial workshop was held to scope the issues faced by trialists when considering whether to use HSDs for trial outcomes. Second, a consultation exercise was undertaken with clinical trials unit (CTU) staff, trialists, methodologists, clinicians, funding panels and data providers. Third, a final discussion workshop was held, at which the results of the consultation were fed back, case studies presented, and issues considered in small breakout groups.

**Results:**

Key topics included in the consultation process were the validity of outcome data, timeliness of data capture, internal pilots, data-sharing, practical issues, and decision-making. A majority of consultation respondents (*n* = 78, 95%) considered the development of guidance for trialists to be feasible. Guidance was developed following the discussion workshop, for the five broad areas of terminology, feasibility, internal pilots, onward data sharing, and data archiving.

**Conclusions:**

We provide guidance to inform decisions about whether or not to use HSDs for outcomes, and if so, to assist trialists in working with registries and other HSD providers to improve the design and delivery of trials.

**Supplementary Information:**

The online version contains supplementary material available at 10.1186/s13063-024-07926-z.

## Background

Healthcare systems data (HSD) refers to health care information, gathered from providers including primary and secondary care, for the delivery of healthcare but not purposely designed for its use in research. Such data are sometimes referred to as routinely collected health data (RCHD). These data may come from administrative, surveillance, registry or audit systems, and may facilitate research, with potential benefits such as a reduction in the burden on patients and health professionals of collecting research-specific data [1].

Between 2013 and 2018, less than 5% of all UK RCTs were granted HSD access from registries [2]. As of 2019, 47% of the 216 in-progress clinical trials in the NIHR Journals library planned to use HSD [3]. Recent estimates show that in 2022, this percentage has increased to 62% [4].

Methodological research priorities for the use of HSD within trials have previously been established through a Delphi study [5]. Stakeholders, including trialists, research funders, regulators, data-providers and the public, identified 40 unique research questions that were ranked in importance via a survey and a virtual consensus meeting. The top seven priorities, in order, relate to data collection method; outcome selection; communication with participants; regulatory approvals; data access and receipt; data quality; and data analysis. A summary is available on the COMORANT study website [6], with full details published [4].

The PRIMORANT study aimed to explore two of the COMORANT methodological research questions by (1) addressing an area of need to establish best practice through methodology work and (2) addressing an area where best practice is clear but not yet implemented through training. This paper describes the work undertaken to address the first of these and focuses on the COMORANT priority question relating to outcome selection at the trial design stage: ‘How should the trials community decide when routinely -collected data for outcomes are of sufficient quality and utility to replace bespoke data collection?’. The aim was to identify issues to be considered before the decision to use HSD for outcome data in a clinical trial is finalised.

## Methods

### Initial workshop

An initial workshop was hosted online on 28th September 2022 and comprised three presentations followed by breakout group discussions. Invitations were distributed in the UK among the COMORANT, Trial Methodology Research Partnership—Health Informatics Working Group (TMRP HI WG), NIHR Methodology Incubator HI subgroup, UK Clinical Research collaboration – Clinical Trials Unit (UKCRC CTU) Network Statistics group, and SPIRIT-Routine lists; 27 people attended. The presentations covered the use of HSD in trials, SPIRIT Extension for trials using routine data and terminology and data integrity. During the breakout groups, it was proposed to discuss, in the context of case studies, how the decision to use HSD was made, alongside lessons learned and relevant guidance. The aim was to identify existing relevant guidance on using HSD data for clinical trial outcomes and to explore areas to consider when using or deciding whether to use HSD.

### Consultation exercise

Based on the six topics identified from the initial workshop, crosschecked for consistency against the existing Medicines and Healthcare Products Regulatory Agency (MHRA) guidance on HSD use in clinical trials for regulatory decisions, [7, 8] 16 questions were developed for the consultation.

The JISC Online Survey tool [9] was used to create, host, and distribute the consultation. All questions were optional, allowing the responder to engage with topics that aligned with their expertise. All responses were provided anonymously. A copy of the consultation questions can be found in Additional file 1.

Between December 2022 and January 2023, the consultation was sent to over 200 individuals, including UK CTU staff, trialists, methodologists, clinicians, funding panels and data providers. Consultation recipients were identified and selected from initial workshop attendees, HTA funding committee members, Chief Investigators (CIs) of RCTs using HSD funded by NIHR and attendees of the SPIRIT Extension report meeting. Recipients were encouraged to distribute the consultation to others with relevant expertise.

### Discussion workshop

The results from the consultation were used to identify issues to be discussed at a face-to-face workshop in March 2023. Respondents to the consultation were asked to note their interest in attending this second workshop, and whether they could present a case study. Findings from the first two stages were summarised descriptively, with free-text responses grouped into topics (initially A-MT and verified by PRW and AF), and presented during the discussion workshop.

Case study presentations were selected by the study team from those offered, based on the range of issues they highlighted and ensuring a diversity of trial designs, trial populations, trial outcomes and data sources. The speakers were asked to prepare a short PowerPoint presentation describing the case study, and the issues related to HSD, alongside their recommendations.

The second part of the workshop focused on the list of issues to consider that arose from the consultation. The participants were divided into six break-out groups and discussed the completeness of the list and generated recommendations for trial teams about points to consider when deciding whether to use HSD for trial outcomes.

## Results

### Initial workshop

**Fig. 1:**
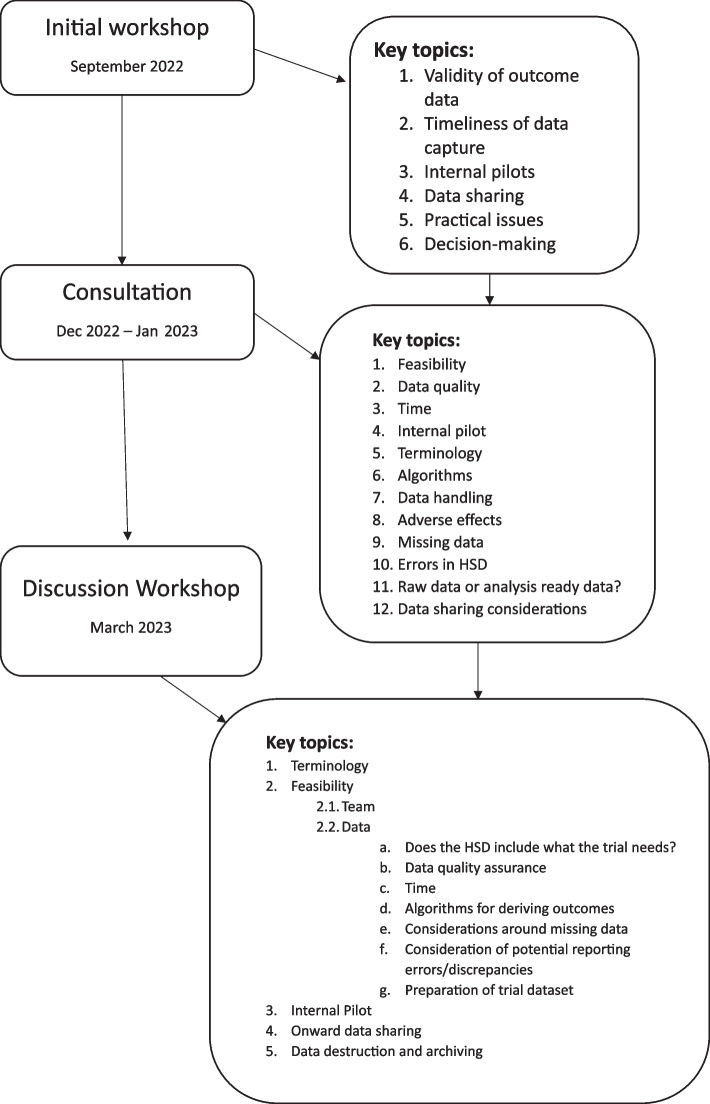
Diagram

The initial workshop was attended by 27 participants. Key topics identified to include in the subsequent consultation process were validity of outcome data, timeliness of data capture, internal pilots, data sharing, practical issues, and decision-making (Fig. 1). The conclusion from the meeting was that the development of practical guidance to be used when considering the use of HSD for outcomes would be helpful.

### Consultation exercise

Responses were received from 82 individuals invited. A majority of responders (*n* = 70) considered that evidence from previous feasibility studies would be sufficient evidence to confirm the validity of outcome data from HSD. Most responders (*n* = 72, 89%) agreed that a Standard Operating Procedure (SOP) for data providers for the handling and resolution of discrepancies in the HSD would be helpful; but fifty responders (61%) considered that such an SOP would not be feasible. In contrast, a template SOP or guidance for trialists was considered feasible by 78 (95%) responders. Further results are shown in Additional file 2.

Many responders (*n* = 64) suggested several elements specifically related to the use of HSD for outcomes to be appropriate for inclusion in trial progression criteria. These included: data availability and completeness; time to access the data; data quality; linkage; the potential for any bias or confounding. Details to be made public by trial teams included: cost of acquiring the data; time needed for each step of the data acquisition and linkage process; and information regarding the quality and validity of data.

Issues to be considered when deciding between using HSD, more traditional data collection through bespoke trial CRFs, or a hybrid approach for collecting outcome data were taken as the starting point for presentations and discussions at the subsequent workshop (Fig. 1).

### Discussion workshop

Invitations were issued to 45 individuals, including the 14 members of the PRIMORANT team, with 35 (78%) able to attend in person, with 5 from the PRIMORANT team. Six case studies and further areas of investigation were presented in 7 talks, and these are summarised in Additional file 3. The key messages across the studies were as follows.

- The need for clear outcome definitions and the use of validated code lists.
- Feasibility assessments can provide assurance about the validity and availability of HSD for outcomes.
- HSD can improve outcome data collection, but challenges include classification; subsequent changes to datasets and linkage; retention and archiving requirements of the clinical trial versus routine data provider; specialist knowledge and resource to analyse hospital episode statistics (HES) data; and adapting traditional data management processes to handle HSD.
- Assessing the utility of HSD against medical records, or through data linkage to other sources, is important in order to understand whether HSD is appropriate for both clinical and health economic outcomes in an individual trial.
- The impact of HSD availability on timing for trial reporting and interim analysis requirements should be considered.
- The volume and types of incomplete data within HSD should be assessed.
- The potential for delay in accessing HSD should be considered against trial timelines.
- Work on demonstrating the integrity and provenance of data is ongoing through collaboration between NHS England (formerly NHS Digital) and HDR UK.

### Feedback from breakout group discussions

Table 1 provides a list of considerations for trialists at the design stage. The content was iteratively discussed and developed during the workshop break-out groups, and subsequently finalised by email.

**Table 1:** Issues to consider. The table describes issues to be considered before the decision to use HSD for collecting outcomes in an RCT is finalised are described here. The aim is to help the trial team make an informed judgement based on an understanding of the suitability of HSD for outcome data in the context of the specific clinical trial, and to build in mitigation, for example including the option to supplement with data directly from participants or sites

Working through the items below may highlight ways trialists can work with HSD providers to improve how such trials are designed and delivered • Trialists should consider additional incurred costs or unanticipated workarounds required during trial, e.g. changes in legislation, delays in data release, periodic renewal of data sharing agreements • Strategies to address uncertainties might include building contingency fund or agreeing phased project plan with funder • Researchers are encouraged to risk assess a broad range of possible scenarios and consider potential mitigation strategies
**(1) Terminology**
• Be aware terminology within data access applications will likely differ between providers; seek clarification/ examples from provider • Ensure awareness of how terms can be interpreted by individuals involved across multiple organisations
**(2) Feasibility**
**2.1 Team**
• Seek to include in trial team: trial operations professionals, data and health specialists with experience of completing data access forms and analysing data from provider/s for relevant health research question • Ideally needs to include individuals who: (1) understand datasets, structure, interpretation and quality; (2) understand how and when data are collected at source; (3) have skills to handle dataset when provided; (4) will undertake statistical and health economic analysis • Where knowledge gaps are identified, seek funding for training and development activities
**2.2 Data**
• Trialists should be aware of how HSD are entered &, coded, QA processes, how data are validated at point of upload and how transferred • Data providers should be approached to provide this information • Trialists should justify the use of healthcare systems datasets in the trial protocol and, in greater detail, in the appropriate section of the Trial Master File (10.1016/S2589-7500(22)00122-4; https://zenodo.org/records/6047155 and Appendix 2 of https://zenodo.org/records/6047938/files/Routine_dataset_justification_template_v1.0_2022-02-15.docx?download=1)
**2.2.a Does the HSD include what the trial needs?**
• Using data provider’s available data dictionary, establish which outcome measures are collected “routinely” • Ascertain costs of data provision • Ascertain data provider timelines for data verification/release • Consider need for repeated data releases and costs relating to data retention • Discuss processes for data linkage if linking to trial cohort and/or multiple data sources are sought • If time and resources permit, interrogate for limitations before deciding to use HSD • Dataset may cover only subset of outcomes relevant to trial question. Consider how other outcome data will be collected, or whether benefit of using single approach to data collection outweighs value of collecting data across multiple sources • Additionally, take into consideration the follow-up outcomes, and their availability form HSD • For registry-based trials, discuss whether registry team could adapt or supplement routine HSD collection to meet trial’s needs without compromising integrity of registry • Whether HSD may be appropriate for aspects of safety reporting depends on clinical trial risk profile. Consider during trial design and define clearly in protocol. Likely to be appropriate in low-risk trials where adverse events are not informing emergent safety profile of treatment • Timeliness of data provision should be considered in relation to safety monitoring plans • Establish whether any precedent, or evidence of public support for accessing these data for research, exists, or alternatively whether issues have arisen previously. Consider trial participants’ needs for understanding of the use of their HSD for outcomes in research and how that may vary according to study populations
**2.2.b Data quality assurance**
• Establish whether provider can provide information regarding data provenance, integrity, and completeness • Understand timeliness of collection of data held by provider, e.g. whether there is lag between site data collection and entry into provider system, or whether data is only released at certain time of year • Understand how provider receives and processes data, and how changes in processing and coding are handled and communicated • Consider what is known, from previous literature, about validity and completeness of outcome data, which may include national audit reports • Assess whether it is realistic to be able to provide funder with accurate idea of HSD data quality at application, or whether it is possible to build in approaches to examine uncertainty during trial
**2.2.c Time**
• Ask provider how long it will take from point of request and then from point of approval to supply specified dataset to trial team • Determine if contract includes binding timelines and decide what is acceptable delay for delivery of data for first occasion and subsequent deliveries • Establish whether this time will reduce if datasets are requested on multiple occasions during trial. Consider in relation to whether interim analyses are planned or when using HSD for monitoring safety outcomes
**2.2.d Algorithms for deriving outcomes**
• Explore whether validated algorithm for deriving outcomes from HSD exists • If not, consider whether to include time to develop and test proposed algorithm, within utility comparison
**2.2.e Considerations around missing data**
• Be aware of timing of data entry processes into HSD resource by clinical teams and data entry clerks, and subsequent availability or missingness, which may vary across sites. For example, within registries outcomes may be entered on annual basis or annual reviews may be delayed • Be aware of how long data may take from local collection into national or collated set, and how long it takes for latter to be released • Discuss whether possible to go back to participating sites to collect missing data • Otherwise consider imputation from other available variables, or other HSD datasets, with collection of extra variables to maximise effectiveness of imputation method. This may be where contingency fund for unanticipated workarounds would be helpful
**2.2.f Consideration of potential reporting errors/discrepancies**
• Discuss mechanism and opportunity for resolution of discrepancies with provider • Ask provider whether they have guidance on range of possible solutions based on experience (e.g. rules of precedence, windows for ‘same dates’, impossible events) • Always cost for managing data queries — could be part of contingency management
**2.2.g Preparation of trial dataset**
• Discuss with provider whether raw data or analysis-ready data will be provided. For example, it may be useful to consider whether trial team will need to do additional analyses for primary analysis, implying raw data more appropriate • However, if the trial team has limited statistical support or only need one or two defined analyses, analysis-ready data might be more appropriate • Cost and time may be a factor — access to analysis-ready data could be more costly or take longer to receive • Additional considerations might be ability to verify derivation of analysis-ready data undertaken by third party. Raw data might be more appropriate here, where the trial team has control over analysis steps provided local statistical expertise exists
**(3) Internal pilot**
• If internal pilot to be undertaken, determine how use of HSD compares to collecting outcome data traditionally, e.g. in terms of sufficiency, timeliness, completeness, and cost-effectiveness. Trial team needs to consider whether setting up trial using both approaches justified in terms of cost and complexity, e.g. by providing added value for health area more widely than individual trial • If internal pilot felt to be valuable and feasible, consider progression criteria to be applied to aspects related to use of HSD
**(4) Onward data sharing**
• Discuss funder’s requirements for onward data sharing and whether provider can approve, considering it can facilitate further research and extend efficiency gains • Ensure issues around onward sharing or subsequent access considered in data sharing agreement/contract as well as any resources involved • Consider prospectively who (in broadest sense, e.g. trial oversight committees, trial team, industry partners, future meta-analysts) needs to see HSD, as raw or aggregated data • Explore legal, ethical and governance responsibilities in advance within appropriate timeframes. There may also be implications for consent forms for the trial, allowing further use of data past initial trial • Ensure any ethical or governance issues regarding the sharing of data from the registry are addressed
**(5) Data destruction and archiving**
• Discuss regulatory requirements for archiving period with data provider, ensuring archiving agreements compliant with clinical trials regulations • Discuss costs associated with holding data for archiving period, and permissions to retain anonymised data, in original or derived format, beyond archive period

## Discussion

To address an agreed methodological research evidence gap prioritised by the research community, we have systematically developed a comprehensive and easy-to-use list of issues to consider when deciding whether to use HSD for trial outcomes. Discussions emphasised the need for careful planning/exploration of the datasets before making the decision. Discussions with funders around phased approaches and contingency planning are recommended.

The FDA has an ongoing Real World Evidence Program, which highlights areas where guidance is needed regarding the quality of HSD data [10]. The CODE-EHR best practice framework for the use of electronic healthcare records in clinical research highlighted several key challenges and paths to improvement that can impact the sustainability of using EHR, by focusing on disseminating aspects about the EHR used [11]. The current work expands on prior literature, creating comprehensive guidance to be considered at the design stage of the clinical trial.

The list also complements other resources available or planned for trialists using HSD for trial outcomes, including MHRA guidance, CTTI guidance [12], and the HDR UK ‘Route Map’ described in Additional file 4. Trialists should be aware of the reviewers of the planned trial protocol, which may differ according to intervention type and bear in mind their standards, if available, eg. MHRA. Forthcoming SPIRIT-Routine guidance is anticipated to highlight some of these issues to be considered in trial protocols [13]. Consideration of the issues described here will also allow trial teams to meet the reporting standards of the CONSORT-Routine guideline [14].

Several areas of potential concern, which are likely to be more commonly encountered, were discussed:

(i) Finding data specialists with experience with HSD can be difficult. If unavailable, identifying appropriate training and funding for this should be built into grant applications, also recognising the increased risk on research delivery and time required.
(ii) Sample datasets are not always available. Early discussion with HSD controllers may be useful, both for them and for the users. AI-generated sample datasets could be developed by the data providers to showcase the dataset, while preventing patient information leakage.
(iii) There are examples of registry trials which supplement core registry data with add-on modules which collect trial-specific outcomes. If this approach is used, data management processes require careful consideration in advance to ensure that the integrity of registry data is not compromised [15].
(iv) When choosing outcome measures, the potential limitations of choosing only those where HSD exists, which may exclude some agreed to be of core importance, e.g. in core outcome sets [16], needs to be considered. Currently, subjective outcome measures, like PROs, are not commonly available from HSD, but over 90% of the RCTs using HSD collected PRO data directly from participants [4]. For PRO data choosing a valid measurement instrument is key, with data utility comparison more challenging.
  Several areas were identified where further work would be helpful.
(v) Validation studies to demonstrate HSD quality are needed in terms of integrity and provenance (Murray 2022) and utility (under review). One question raised was whether data providers should be responsible for providing information about the validity of the data they provide. Expansion of the work to demonstrate integrity and provenance of data [17, 18] to cover more providers will be useful.
(vi) Examples of helpful discussions with research funders were given, whereby phased feasibility studies to assess uncertainties related to HSD were agreed. A point for further discussion with funders is whether a different costing model should be applied to access data for feasibility and pilot studies. It was considered helpful to explore this concept with funders and HSD providers, to see how it might be potentially supported.

Strengths of this work include the range of stakeholders engaged, and the breadth of examples and case studies discussed. The responses to the consultation allowed the exploration of a range of potential areas for consideration that mapped onto issues across the lifecycle of the trial and covered topic areas that were likely to be relevant to the range of disciplines and roles involved in trial design. Limited representation of funders and data providers, both public and from industry, at the discussion workshop, is recognised as a limitation; however, planned dissemination activities will be aimed at greater engagement, with potential for future revisions to the list of issues to consider. The main focus of this work was on UK practice and datasets, although some of the findings may be considered to be generalisable outside the UK.

The focus of the PRIMORANT study was on issues to consider during the design phase of a clinical trial. It is important to note however that there are other aspects of conduct and reporting in relation to using HSD for trial outcomes. For example, algorithms used within trials should be well-documented to enhance reproducibility. Code list and data fields provided may change over time, so algorithms will need to change, and those changes will also need to be documented. If data are sourced from multiple providers, consistency of coding across the datasets should be checked and reconciliation clearly documented. Code lists and/or algorithms should be made publicly available to improve efficiency for future researchers, for example in the HDR UK phenotype library.

## Conclusion

In summary, the issues identified here should strengthen the decision-making process for trialists when considering the use of HSD for trial outcomes. The work should also inform discussions with funders to build in mitigation (e.g. include an option to supplement with data directly from participants or sites) and allow for additional costs that could be incurred or unanticipated workarounds required (e.g. for changes in legislation, delays in data release, periodic renewal of data sharing agreements), as well as discussions with HSD-providers about how to improve the design and delivery of trials using HSD.

### Supplementary Information

**Additional file 1.** Consultation. Contains the printable version of the consultation that was sent online.

**Additional file 2.** Consultation results. Contains the results of the consultation in table format.

**Additional file 3.** Case studies presented at the final workshop. Contains the summaries of the case-studies presented the final workshop.

**Additional file 4.** Diagram. Presents the key topics identified at each of the workshops and consultation.

## Data Availability

Not applicable.

## Abbreviations

A&E: Accidents and emergency
CAG: Confidentiality advisory group
COPD: Chronic obstructive pulmonary disease
CTTI: Clinical Trials Transformation Initiative
CTU: Clinical trials unit
DfE: Department for education
DMC: Data monitoring committee
EHR: Electronic health record
FDA: Food and Drug Administration
HER: Electronic health records
EMIS: Egton medical information systems
HDR: Health data research
HES: Hospital episode statistics
HRA: Health research authority
HSD: Healthcare systems data
ICD-10: International classification of diseases, 10th revision
MHRA: Medicines and healthcare products regulatory agency
NHS: National health service
NIHR: National Institute for health and care research
ONS: Office for national statistics
PRO: Patient reported outcome
PROMs: Patient-reported outcome measures
PSA: Prostate-specific antigen
QA: Quality Assurance
QoL: Quality of life
RCHD: Routinely collected health data
RCT: Randomised controlled trial
REC: Research ethics committee
SOP: Standard operating procedures
TARN: Trauma audit and research network
UKPDS: UK prospective diabetes study
