# Supplementary material for: Using healthcare systems data for outcomes in clinical trials: issues to consider at the design stage
Source: Trials. 2024 Jan 29;25:94. doi: 10.1186/s13063-024-07926-z (PMC10823676; doi:10.1186/s13063-024-07926-z)
Supplement: Supplementary file 1 — Additional file 1. Consultation. Contains the printable version of the consultation that was sent online. [file 13063_2024_7926_MOESM1_ESM.pdf]

PRIMORANT

Page 1

This consultation relates to the question: "***How should the trials community decide when routinely collected data for outcomes is of sufficient quality and utility to replace bespoke data collection?***".

A note about terminology: By '*routinely collected data'* (RCD) we mean data that has not been collected with a specific research question in mind. In the questions below, we refer to the MHRA guidance on the use of real-world data (RWD) in clinical studies to support regulatory decisions.

Topic 1 - Validity of outcome data

The MHRA guidance states '*It is recommended that an observational feasibility study is conducted by the sponsor to assess the recent capture of study variables prior to undertaking a RWD study' and 'It is expected that the validity of the RWD that are intended to be used in the study is formally documented and approved by the sponsor before the study protocol is published or submitted to the MHRA.*'

What evidence would you consider sufficient to establish the reliability/validity of RCD for trial outcomes? (tick as many as appropriate)

- [ ] Evidence from a previous feasibility study
- [ ] Evidence of previous use in a research study
- [ ] Expert opinion from someone familiar with the data source
- [ ] Algorithms to derive outcomes published
- [ ] Other - please specify

If you selected Other, please specify:

Topic 2 - Timeliness of data capture for target outcomes and frequency (actual and expected) of data receipt

The MHRA guidance recommends that you should consider the following aspects of RWD: '*Is the time between the occurrence of events and availability of the data to the study team suitable for the usage of the data in the study? How soon will data be able to be analysed after last patient, last visit? If used for monitoring adverse events, what impact would the availability of data have on the suitability of the database?*'

In relation to these considerations, are there situations where you have discounted/would discount using RCD?

The MHRA guidance states that '*Processes should exist for the resolution of discrepancies and communication of issues identified by later processing*'.

Would a common SOP for resolution of discrepancies for data providers be helpful?

- Yes
- No

Please explain why

Would a common SOP for (all) data providers be at all feasible?

- Yes
- No

Please explain why

Would a template/SOP/guidance for trialists be (any) more feasible?

- Yes
- No

Please explain why

What do you think is key to include in such a common SOP?

Topic 3 - Internal pilot to look at validity of routine data for trial outcomes

It can be challenging to access relevant RCD in advance of a study being funded. There are advantages and disadvantages of receiving RCD partway through the trial vs at the end of the trial. Considerations include: the cost to download data periodically; the amount of work needed for every download and its management; the benefits from understanding the data structure, content, quality prior to final analysis.

Are you aware of trials using RCD for outcomes where an internal pilot would be/would have been helpful?

- Yes
- No

If you selected Yes, please specify:

What Stop/Go/Amend progression criteria might you consider in such an internal pilot?

Page 2

Topic 4 Provision of RCD and onward sharing of trial outcome data

Do you have any comments to make about issues related to the following aspects of data sharing? You may wish to answer in general terms, or in relation to specific data providers.

- ☐ Data linkage
- ☐ Data sharing agreements
- ☐ Whether raw data or an analysis-ready dataset will be provided
- ☐ Approach to changes in coding systems
- ☐ Onward data sharing ability, e.g. for a safety monitoring report
- ☐ Level of anonymisation
- ☐ Other – please specify

Data linkage

Data sharing agreements

Whether raw data or an analysis-ready dataset will be provided

Whether raw data or an analysis-ready dataset will be provided

Onward data sharing ability, e.g. for a safety monitoring report

Level of anonymisation

Other

Topic 5 - Lack of published detail about practical issues related to use of RCD for trial outcomes

Which practical/logistical aspects of using routine data would be most helpful to publish, e.g. time to receive data, cost, processing challenges/guidance etc? For example: https://trialsjournal.biomedcentral.com/articles/10.1186/s13063-017-2135-9

Topic 6 - Decision-making

What decision criteria would you use to decide between using RCD for trial outcomes vs using more traditional ways of collecting data for trial outcomes vs RCD being supplemented by more traditional data collection methods?

Page 3

General questions

Are there other important considerations to inform the decision to use RCD for trial outcomes, which have not been described above?

Do you have a case study related to any of the topics above that you would be willing to share here or at the meeting in March? This may be where you are currently assessing feasibility of using routine data for outcomes, or it may be a trial which is ongoing but where you have learnt some lessons to share. Please provide some details.

Please provide any references you are aware of that discuss any of the topics above.

Workshop attendance

Would you like to be considered for a place at the workshop to develop the guidance on Tuesday 7th March, 10am-4pm (approx.) in London? If so, please provide:

Your name

Your email address

Please enter a valid email address.

Final page

Thank you for completing this consultation.

If you entered your details for the workshop attendance, we will be in touch soon.
