# Supplementary material for: Using healthcare systems data for outcomes in clinical trials: issues to consider at the design stage
Source: Trials. 2024 Jan 29;25:94. doi: 10.1186/s13063-024-07926-z (PMC10823676; doi:10.1186/s13063-024-07926-z)
Supplement: Supplementary file 2 — Additional file 2. Consultation results. Contains the results of the consultation in table format. [file 13063_2024_7926_MOESM2_ESM.docx]

Additional file 2 Results from the consultation

| **1. Validity of outcome data** | | | |
| --- | --- | --- | --- |
| **What evidence would you consider sufficient to establish the reliability/validity of RCD for trial outcomes?** | | | |
|  | Evidence from previous feasibility study | | 70 (85.4%) |
|  | Evidence from previous use in a research study | | 54 (65.9%) |
|  | Expert opinion from someone familiar with the data source | | 39 (47.6%) |
|  | Algorithms to derive outcomes published | | 38 (46.3%) |
|  | Other | | 20 (24.4%) |
|  | Validation study | | 4 (5%) |
|  | Feasibility study | | 2 (2%) |
|  | Any empirical study | | 2 (2%) |
|  | Document from data provider | | 2 (2%) |
|  | Previous use evidence | | 2 (2%) |
| **2. Timeliness of data capture for target outcomes and frequency (actual and expected) of data receipt** | | | |
| **Are there situations where you have discounted/would discount using RCD?** | | | |
|  | When aware of the delay in getting the data | | |
|  | Safety of treatment need careful monitoring | | |
|  | Cost efficiency | | |
|  | Insufficient data or quality of data | | |
|  | Insufficient information about the data | | |
| **Would a common SOP for resolution of discrepancies for data providers be helpful?** | | | |
|  | Yes | | 72 (86.7%) |
|  | No | | 11 (13.3%) |
| **Would a common SOP for (all) data providers be at all feasible?** | | | |
|  | Yes | | 27 (35.1%) |
|  | No | | 50 (64.9%) |
| **Would a template/SOP/guidance for trialists be (any) more feasible?** | | | |
|  | Yes | | 78 (95.1%) |
|  | No | | 4 (4.9%) |
| **What do you think is key to include in such a common SOP?** | | | |
|  | Resolution of discrepancies | | |
|  | Agreeing response timeliness | | |
|  | Data handling & algorithms | | |
|  | Missing data | | |
|  | Data quality assurance (including any data linkage) | | |
|  | Terminology | | |
|  | Considerations – questions to consider | | |
|  | Error handling | | |
|  | Adverse effects | | |
| **3. Internal pilot to look at validity of routine data for trial outcomes** | | | |
| **Are you aware of trials using HSD for outcomes where an internal pilot would be/would have been helpful?** | | | |
|  | Yes | | 35 (42.7%) |
|  | No | | 47 (57.3%) |
| **What Stop/Go/Amend progression criteria might you consider in such an internal pilot?** | | | |
|  | What data is available? | | |
|  | Missing data | | |
|  | Time to access the data | | |
|  | Quality of data | | |
|  | Successful linkage | | |
|  | Bias | | |
| **4. Provision of RCD and onward sharing of trial outcome data** | | | |
| **Additional issues related to the following topics:** | | | |
|  | Data sharing agreements | 34 (55.7%) | |
|  | Whether raw data or analysis-ready dataset will be provided | 30 (49.2%) | |
|  | Onward data sharing ability | 25 (41%) | |
|  | Data linkage | 23 (37.7%) | |
|  | Level of anonymisation | 21 (34.4%) | |
|  | Approach to changes in coding systems | 17 (27.9%) | |
|  | Other | 5 (8.2%) | |
| **5. Lack of published detail about practical issues related to use of RCD for trial outcomes** | | | |
| **Which practical/logistical aspects of using routine data would be most helpful to publish?** | | | |
|  | Cost | | |
|  | Time – including approximation for each step of the process | | |
|  | Processing challenges and methodology | | |
|  | Quality of data – including data validity and Missing data | | |
|  | Case studies and FAQs | | |
|  | Feasibility and validity studies | | |
| **6. Decision-making** | | | |
| **What decision criteria would you use to decide between using HSD for trial outcomes vs using more traditional ways of collecting data for trial outcomes vs HSD being supplemented by more traditional data collection methods?** | | | |
|  | Cost and resources needed | | |
|  | Outcomes of Interest | | |
|  | Quality of data | | |
|  | Time to access the data | | |
|  | Feasibility | | |
|  | Transparency of data | | |
|  | Patient burden | | |
|  | Linkage | | |
|  | Difficulty of process | | |
|  | Bias | | |
