# Supplementary material for: Using healthcare systems data for outcomes in clinical trials: issues to consider at the design stage
Source: Trials. 2024 Jan 29;25:94. doi: 10.1186/s13063-024-07926-z (PMC10823676; doi:10.1186/s13063-024-07926-z)
Supplement: Supplementary file 3 — Additional file 3. Case studies presented at the final workshop. Contains the summaries of the case-studies presented the final workshop. [file 13063_2024_7926_MOESM3_ESM.docx]

**Additional file 3 Presentations at the Final Workshop**

**Talk 1** (Jennifer Quint): Expert opinion from someone familiar with the data source

HSD are increasingly used for research purposes, not only for observational studies but also for pragmatic trials. Their use is increasingly considered to provide additional information to end points for trials or extending the duration of a trial for long term follow up, e.g., to explore mortality. These data have many strengths including large population sizes, a wide breadth of health-related information and are more generalisable to wider populations.

**Key messages:** One important aspect of HSD use is quality, in particular validity of variables of interest, such as study exposures and outcomes. Prospective studies, including trials, have clear definitions of study endpoints that are comparable between studies. Studies using HSD do not always have clear clinical definitions, and some disease endpoints e.g. exacerbations of COPD have been defined across studies in more than one way (1). Poor quality definitions can lead to identification of incorrect variables and misclassification of events, leading to varying study results including over or underestimation of disease prevalence and limited generalisability of study findings (2). Validation of study exposure and outcome definitions, wherever possible, is essential to ensure robust, comparable outcomes.

Transparency around how outcomes have been created is also key (3). Although there are clear methodologies for code lists creation, and repositories for outcomes that have been created, these are not always used. In addition, different databases use different coding ontologies, and it is not always easy to map between them.

**Talk 2** (Michael Robling): Assessing the feasibility of using routine healthcare systems data to follow up the Building Blocks trial cohort

**Study description:** The research team conducted a trial of specialist home visiting with 1618 first-time mothers in England who had been followed up to 24 months postpartum (4). However, intervention benefits were hypothesised to be observed for both mother and child over a much longer time-period, similar to those reported in earlier North American trials. Although the trial had itself used HSD (in addition to self-reported data) with participant consent, ethical approval for the trial did not extend to such long-term follow-up. Therefore, designed a cohort study to follow families for a further five years using an opt-out linkage model supported by section 251 approval and linking to new data sets from education and social care, in addition to healthcare. The researchers agreed with their funder (NIHR Public Health Research Programme) to conduct a two-phase feasibility assessment of their new cohort study to address some uncertainties in their planned approach. The first phase addressed parameters from the trial itself such as adequacy of intervention fidelity and absence of harms attributable to the intervention. These criteria were evidenced by the end of the trial.

The second phase addressed features of the data linkage model and comprised three steps. The first step was acceptability and consequences of the dissent model for a cohort who had previously consented to participate in a trial. This dissent approach was reviewed and informed by a lay advisory group and involved multiple routes for women to be informed about the new study (e.g., text, email, letter) to enable dissent to be registered where required. The approach satisfied HRA (Confidentiality Advisory Group and Research Ethics Committee), and data provider requirements. Of 1,545 mothers contacted, only eight (<0.01%) mothers dissented, retaining the validity of the original cohort. Steps 2 and 3 involved data access requests to match and retrieve a portion of what would be the full period of intended follow-up. Step 2 assessed record linkage adequacy, specifically the number and proportion of participant identifiers matched to records by each data centre (NHS Digital and Department for Education (DfE)). Match rates were high for both providers (e.g., 98% for NHS Digital data, 90% for DfE data) while reflecting the differing models for data linkage for each provider. Step 3 assessed whether key outcomes (such as being classified as a child in need) could be adequately derived from supplied data. For all fields retrieved, an impact assessment was implemented to clarify the field’s role in analysis, number of records retrieved, notes about missingness and impact on planned analysis.

**Key messages:** While some planned analyses of secondary outcomes were shown to be potentially affected by validity of data retrieved (e.g., due to missingness), the feasibility assessment provided assurance about the validity of retrievable data for the primary and most other secondary outcomes.

**Talk 3** (Alexandra Wright-Hughes): Assessing the feasibility, reliability and accuracy of using routine data for mental health outcomes in the SHIFT trial

**Study description:** The SHIFT trial compared family therapy with treatment as usual in 832 young people seen after episode/s of self-harm across 40 child and adolescent mental health units in England (5). The primary outcome was repetition of self-harm leading to hospital attendance within 18 months, collected objectively from medical records. Initially this data was collected via a traditional approach by trial researchers who visited hospitals in ‘SHIFT areas’ to manually interrogate local medical records. However, challenges with this approach included obtaining and facilitating access to multiple hospital trusts, intensive resource requirements and restricted regional coverage.

To overcome these issues, the feasibility, reliability and accuracy of HSD from Hospital Episode Statistics (HES) via NHS Digital was explored (6). Following a pilot linkage to A&E attendances and admissions, 341 complete hospital episodes were derived, compared to 139 retrieved via the initial approach by that time. Based on A&E patient group and admission ICD-10 codes 61 (18%) and 69 (20%) episodes were classified as related and not related to self-harm. respectively, leaving 211 (62%) unclassified. The trial proceeded with a hybrid approach, using HSD to identify attendances, followed by targeted researcher follow-up of unclassified (largely A&E) attendances. By the end of the trial, targeted researcher data collection took place for 54% of attendances and HES was found to underestimate self-harm attendances by 37% (95% CI: 33%, 42%) based on ~161 unclassified attendances in HES of ~433 known self-harm attendances.

**Key messages:** Whilst this study found it was possible to incorporate HSD to improve outcome data collection, challenges included classification; lengthy processes to gain approval, subsequent changes to datasets and linkage; retention and archiving requirements of the clinical trial versus routine data provider; specialist knowledge and resource to analyse HES data; and adapting traditional data management processes to handle HSD. Related recommendations included incorporating pilot data to ensure HSD can be obtained and outcomes derived, to verify HSD against other sources, to carefully plan the timing of HSD downloads against annual data updates, time-lags and trial reporting requirements, and to consider data provider consent, data flow, and security requirements at trial set up.

**Talk 4** (Joanna Thorn): The CAP trial

**Study description:** The CAP trial is a cluster randomised trial in England and Wales seeking to determine the effectiveness and cost-effectiveness of Prostate Specific Antigen (PSA) testing for prostate cancer (7). Men aged 50 to 69 were recruited from eight centres between 2001 and 2009 to receive either an invitation to undergo a one-off PSA test (intervention) or usual care. The trial found no evidence of a significant difference between the arms in prostate cancer mortality after a median of 10 years of follow-up (7). At trial outset, secondary care resource-use data were collected via a medical note review process for all men who died either of or with prostate cancer, with the intention of informing an economic evaluation. The process was meticulously carried out by highly trained researchers, requiring significant resource input. The use of HSD was therefore explored as an alternative, focusing on HES, which contains records of all hospital events in NHS hospitals in England. However, a protracted discussion process with NHS Digital, the body with responsibility for managing HES data, meant that the data were not made available until some three years after the first formal data request submission.

**Key messages:** Given that the HES dataset was not compiled for research purposes, it was important to assess whether it was valid to use in this way, and the detailed medical note review data collected earlier in the trial facilitated two validation studies to fulfil this aim. The validity of the admitted patient care dataset was explored by comparing monthly costs derived from medical note review and HES for men in the final year of life (8). The outpatient dataset was tested by matching appointments recorded in the medical note review with appointments captured in HES (9). Both studies identified errors in the HES datasets. For admitted patient care, episodes were identified via medical note review for seven men (of 292, 2.4%) who had no hospital events recorded in HES, while for the outpatient dataset, 215/370 men (58.1 %) had one or more appointments identified in the medical note review process that were not recorded in HES. The studies concluded that HSD were adequate for research purposes, with some caveats about particular limitations (for example, the outpatient dataset contained insufficient information to identify reasons for appointments). However, until the data can be reliably supplied in a timely fashion, the use of HSD will remain sub-optimal.

**Talk 5** (Ly-Mee Yu): The SuMMiT-D trial

**Study description:** SuMMiT-D is a multicentre trial conducted in primary care to determine the effectiveness and cost-effectiveness of mobile phone-based text messages, developed using behaviour change techniques, to promote self-management of medications for reducing cardiovascular risk in people with type 2 diabetes versus usual care. An external feasibility study was conducted, which recruited 209 participants, to inform the design and delivery of the main trial (1039 participants recruited). Details about the trial can be found elsewhere (10, 11). The primary outcome is 10-year percentage risk score of coronary heart disease at 12 months from randomisation, defined as a composite cardiovascular outcome based on the equation used for the UK Prospective Diabetes Study (UKPDS) risk engine (12). Clinical measurement data used for the calculation of the primary outcome, such as HbA1c, systolic blood pressure, HDL cholesterol, and total cholesterol were collected from routinely collected healthcare data. These data were manually extracted from medical records in the feasibility trial and through data linkage using Egton Medical Information Systems (13) in the main trial.

**Key messages:** Both methods of data extraction captured clinical measures at 12 months from randomisation. The majority of measures extracted were similar in both methods, except for total cholesterol which had substantial missing data from notes review. This resulted in only 56 of participants in the feasibility study (n=209) having measures available for the primary outcome. In comparison, data obtained directly from EMIS provided at least 95% of participant’s data in the main trial (n=1039) for the primary outcome. It was not clear why the total cholesterol measure was not available in the manual extract. One explanation could be due to discrepancy in how the data were extracted by different team members. The feasibility trial provided an opportunity to better understand data availability when using routinely collected health data. The use of EMIS for data linkage has increased the efficiency of data collection and high proportion of data extracted. However, the disadvantages are limitation to EMIS-enabled practices in primary care being included in a trial and the cost of data linkage. The latter can be considered having a different pricing model for feasibility or smaller studies.

**Talk 6** (Marion Campbell): Insights from the use of HSD in the UK-REBOA trial

**Study description:** UK-REBOA was a multi-centre, Bayesian trial comparing standard care plus Resuscitative Endovascular Balloon Occlusion of the Aorta (REBOA) versus standard care alone for the management of exsanguinating torso haemorrhage in 16 major trauma centres in England. It was designed to be registry-enabled with data being obtained from the Trauma Audit Research Network (TARN) national registry and NHS Digital (with HES data for hospital resource use and mortality data through Office for National Statistics datasets generated from civil registrations) in addition to some case report form data. All major trauma centres in England are required to submit data to TARN and data had been previously reported to be of high quality and well completed (see tarn.ac.uk). TARN collect demographic, injury, treatment, and outcome data and additionally – through a third-party provider – patient-reported outcome measures (PROMs), including EQ-5D-5L to assess quality of life (QoL). Data are uploaded to TARN from clinical sites.

**Key messages:** Whilst efficient, using HSD raised a number of issues for the trial. Firstly, data availability impacted completeness of data available for Data Monitoring Committee decision-making. Two interim analyses were planned over the course of the trial. Delays in receiving patient characteristics, treatment and mortality data meant that the DMC often did not have fully complete data to inform their decision making. Secondly, issues arose with the third-party provider of the quality of life (QoL) data. Following the first linkage run, it became clear that the QoL results collected by the third-party provider were incomplete. The research team then pivoted to collect QoL data directly from the participant prior to their discharge, and subsequently at approximately six months after randomisation by telephone. Both these issues required workarounds (and resources) that had not been anticipated. Additionally, there were major issues gaining approval for access to data held at NHS Digital (although this was exacerbated as the trial was conducted in the emergency setting where initial enrolment took place without consent). Repeated turnover of NHS Digital case workers resulted in the study progress being further hampered, often with different issues raised as the trial was transferred between new members of the NHS Digital team (approval was finally given after ~5 years of interaction). Early scenario planning for possible issues may be a useful strategy to consider for trialists considering the use of HSD and potentially the integration of contingency in the trial budget to address possible problems.

**Talk 7** (Matt Sydes): Agreement and completeness of routine versus trial-specific patient outcome data

Health Data Research UK (HDR UK) (14) is the national institute of health data science with a mission to unite the nation’s health data to enable discoveries that improve people’s lives. Facilitating clinical trials is a key focus for HDR UK’s second five-year quinquennium period (2023-2028) through the Transforming Data for Trials programme established within Pillar 3: Useable Data of the Infrastructure and Services programme. The use of Healthcare Systems Data (HSD), data already collected as part of the delivery of healthcare, has the capacity to transform the way clinical trials are done but there are many specific challenges to address when using such data in a clinical trial setting. The Transforming Data for Trials programme aims to provide tools and resources for trial teams to recognize the potential for, and be able to use, HSD to transform recruitment and follow-up to trials, and to address barriers to the acceptance of such approaches. This requires new tools and new expertise, both within trials units but also for the consumers of trial results, where it is vital that trial results are accepted.

Capability for trials units will be expanded through the development of a “Route Map” for trials using HSD, modelled on the NIHR toolkit. Courses and training will be developed and de-siloing the current pockets of expertise will be facilitated through effective knowledge transfer and the facilitation of mentoring links. A searchable Case Study Catalogue will be developed to showcase trials successfully using HSD and to share re-usable methods and lessons learned. A new Trials Stakeholder Prioritisation Forum is being initiated; this will be the first group to explicitly draw together, on a regular basis, academic and industry trials researchers, data providers regulators, ethics committees and funders from all 4 UK nations with the purpose of prioritising how the current blockers to using HSD in trials should be, and are being, addressed. Work on demonstrating the integrity and provenance of data, started with NHS England (formerly NHS Digital) in HDR UK’s first 5 years, (15, 16) will continue and has been expanding to other data providers. To use HSD, its suitability to replace aspects of trial-specific data collection must be demonstrated. This can be done through data utility comparisons in which trial outcomes derived from HSD are compared to ‘gold-standard’ adjudicated trials outcomes. A small number of these studies are already published, including (17-19), and further studies will be supported. The programme draws on leadership across the UK in a collaboration between the University of Dundee, University of Cardiff, University College London and the University of Oxford. Working with HDR UK, patient and public involvement will be integrated throughout, and close links with the MRC-NIHR Trials Methodology Research Partnership will be maintained.

**References**

1. Rothnie KJ, Müllerová H, Hurst JR, Smeeth L, Davis K, Thomas SL, et al. Validation of the recording of acute exacerbations of COPD in UK primary care electronic healthcare records. PLoS one. 2016;11(3):e0151357.

2. MacRae C, McMinn M, Mercer SW, Henderson D, McAllister DA, Ho I, et al. The impact of varying the number and selection of conditions on estimated multimorbidity prevalence: A cross-sectional study using a large, primary care population dataset. PLoS Medicine. 2023;20(4):e1004208.

3. Langan SM, Schmidt SA, Wing K, Ehrenstein V, Nicholls SG, Filion KB, et al. The reporting of studies conducted using observational routinely collected health data statement for pharmacoepidemiology (RECORD-PE). bmj. 2018;363.

4. Lugg-Widger F, Cannings-John R, Angel L, Moody G, Segrott J, Kenkre J, et al. Assessing the impact of specialist home visiting upon maltreatment in England: a feasibility study of data linkage from a public health trial to routine health and social care data. Pilot and feasibility studies. 2018;4(1):1-11.

5. Cottrell DJ, Wright-Hughes A, Collinson M, Boston P, Eisler I, Fortune S, et al. Effectiveness of systemic family therapy versus treatment as usual for young people after self-harm: a pragmatic, phase 3, multicentre, randomised controlled trial. The Lancet Psychiatry. 2018;5(3):203-16.

6. Wright-Hughes A, Graham E, Cottrell D, Farrin A. Routine hospital data–is it good enough for trials? An example using England’s Hospital Episode Statistics in the SHIFT trial of Family Therapy vs. Treatment as Usual in adolescents following self-harm. Clinical trials. 2018;15(2):197-206.

7. Martin RM, Donovan JL, Turner EL, Metcalfe C, Young GJ, Walsh EI, et al. Effect of a low-intensity PSA-based screening intervention on prostate cancer mortality: the CAP randomized clinical trial. Jama. 2018;319(9):883-95.

8. Thorn JC, Turner EL, Hounsome L, Walsh E, Down L, Verne J, et al. Validating the use of Hospital Episode Statistics data and comparison of costing methodologies for economic evaluation: an end-of-life case study from the Cluster randomised triAl of PSA testing for Prostate cancer (CAP). BMJ open. 2016;6(4):e011063.

9. Thorn JC, Turner E, Hounsome L, Walsh E, Donovan JL, Verne J, et al. Validation of the hospital episode statistics outpatient dataset in England. Pharmacoeconomics. 2016;34:161-8.

10. Farmer A, Allen J, Bartlett K, Bower P, Chi Y, French D, et al. SuMMiT-D Collaborative Group Supporting people with type 2 diabetes in effective use of their medicine through mobile health technology integrated with clinical care (SuMMiT-D Feasibility): a randomised feasibility trial protocol. BMJ Open. 2019 Dec 29; 9 (12): e033504. doi: 10.1136/bmjopen-2019-033504.

11. Farmer A, Jones L, Newhouse N, Kenning C, Williams N, Chi Y, et al. Supporting People With Type 2 Diabetes in the Effective Use of Their Medicine Through Mobile Health Technology Integrated With Clinical Care to Reduce Cardiovascular Risk: Protocol for an Effectiveness and Cost-effectiveness Randomized Controlled Trial. JMIR research protocols. 2022;11(2):e32918.

12. Stevens RJ, Kothari V, Adler AI, Stratton IM, Holman RR, Group UKPDS. The UKPDS risk engine: a model for the risk of coronary heart disease in Type II diabetes (UKPDS 56). Clinical science. 2001;101(6):671-9.

13. EMIS Health.

14. Health Data Research UK (HDR UK) [Available from: <https://www.hdruk.ac.uk/>.

15. Murray ML, Love SB, Carpenter JR, Hartley S, Landray MJ, Mafham M, et al. Data provenance and integrity of health-care systems data for clinical trials. The Lancet Digital Health. 2022;4(8):e567-e8.

16. Murray ML, Pinches H, Mafham M, Hartley S, Carpenter J, Landray MJ, et al. Use of NHS Digital datasets as trial data in the UK: a position paper. 2022.

17. Macnair A, Nankivell M, Murray ML, Rosen SD, Appleyard S, Sydes MR, et al. Healthcare systems data in the context of clinical trials–A comparison of cardiovascular data from a clinical trial dataset with routinely collected data. Contemporary Clinical Trials. 2023;128:107162.

18. Love SB, Kilanowski A, Yorke-Edwards V, Old O, Barr H, Stokes C, et al. Use of routinely collected health data in randomised clinical trials: comparison of trial-specific death data in the BOSS trial with NHS Digital data. Trials. 2021;22:1-10.

19. Harper C, Mafham M, Herrington W, Staplin N, Stevens W, Wallendszus K, et al. Comparison of the accuracy and completeness of records of serious vascular events in routinely collected data vs clinical trial–adjudicated direct follow-up data in the UK: secondary analysis of the ASCEND randomized clinical trial. JAMA Network Open. 2021;4(12):e2139748-e.
