# Supplementary material for: Using healthcare systems data for outcomes in clinical trials: issues to consider at the design stage
Source: Trials. 2024 Jan 29;25:94. doi: 10.1186/s13063-024-07926-z (PMC10823676; doi:10.1186/s13063-024-07926-z)
Supplement: Supplementary file 4 — Additional file 4. Diagram. Presents the key topics identified at each of the workshops and consultation. [file 13063_2024_7926_MOESM4_ESM.docx]

**Key topics:**

1. Validity of outcome data
2. Timeliness of data capture
3. Internal pilots
4. Data sharing
5. Practical issues
6. Decision-making

**Key topics:**

1. Feasibility
2. Data quality
3. Time
4. Internal pilot
5. Terminology
6. Algorithms
7. Data handling
8. Adverse effects
9. Missing data
10. Errors in HSD
11. Raw data or analysis ready data?
12. Data sharing considerations

**Key topics:**

1. Terminology
2. Feasibility
   1. Team
   2. Data
      1. Does the HSD include what the trial needs?
      2. Data quality assurance
      3. Time
      4. Algorithms for deriving outcomes
      5. Considerations around missing data
      6. Consideration of potential reporting errors/discrepancies
      7. Preparation of trial dataset
3. Internal Pilot
4. Onward data sharing
5. Data destruction and archiving

Discussion Workshop

March 2023

Consultation

Dec 2022 – Jan 2023

Initial workshop

September 2022
